# A comparative study of the gut microbiome and fecal metabolome in hypertensive patients from middle-temperate and tropical cities of China: Daqing and Haikou

**DOI:** 10.3389/fmicb.2026.1801806

**Published:** 2026-05-22

**Authors:** Shuyue Liu, Xianyun Luo, Jing Zhou, Lingqi Wang, Rui Li, Ziyue Luo, Na Li, Sha Xiao, Ping Zhang

**Affiliations:** 1 Key Laboratory of Tropical Translational Medicine of Ministry of Education, School of Public Health, Hainan Academy of Medical Sciences, Hainan Medical University, Haikou, Hainan, China; 2 Department of Cardiology, Geriatric Hospital of Hainan, Haikou, Hainan, China; 3 Department of Laboratory Diagnosis, Fifth Affiliated Hospital of Harbin Medical University, Daqing, Heilongjiang, China

**Keywords:** gut microbiome, hypertension, metabolome, middle-temperate zone, tropical zone

## Abstract

**Background:**

Geographic variations in climate and lifestyle may be associated with hypertension (HTN) through alterations in the gut microbiota and its metabolites. This study aimed to comparatively analyze the gut microbiome and fecal metabolome of hypertensive patients from two Chinese cities characterized by distinct climatic conditions: Daqing (middle-temperate climate) and Haikou (tropical climate). The objective was to identify gut microbial and metabolic characteristics associated with geographic differences and to provide insights into HTN prevention and management.

**Methods:**

A cross-sectional study was conducted between May and December 2024, involving hypertensive patients from Daqing and Haikou. Fecal samples were collected from 28 hypertensive patients in Daqing (DQ group) and 32 in Haikou (HK group), and analyzed using shotgun metagenomic sequencing and untargeted metabolomics.

**Results:**

Differences in microbial composition and metabolite profiles were observed between the two groups. Using ALDEx2 analysis at the genus level, 34 genera were identified as differentially abundant between the DQ and HK groups. After adjusting for potential confounding variables, including age, body mass index, smoking, and drinking status, 6 genera remained significantly associated with geographic grouping. A logistic regression model based on these genera achieved an area under the curve (AUC) of 0.8069, with *Pseudescherichia* showing the highest individual discriminatory performance (AUC = 0.7925). Functional analysis suggested that pathways such as xylene degradation and biofilm formation were relatively reduced in the DQ group. Metabolomic analysis identified 38 differentially abundant metabolites, including 15-hydroxyeicosatetraenoic acid (15-HETE), 7α,25-dihydroxycholesterol, the putative metabolite (3-hydroxypentadecanoyl) lysine, and ginsenoside Rg3. Dysregulated pathways were mainly involved in glycerophospholipid metabolism, ABC transporters, and choline metabolism. Correlation analysis revealed potential associations between differential microbes and metabolites.

**Conclusion:**

Distinct gut microbiome and metabolome profiles were observed between hypertensive patients from the two geographic regions. These findings suggest potential associations between environmental factors and host-microbiome-metabolite interactions.

## 1 Introduction

Hypertension (HTN) is a complex and chronic disease with a multitude of symptoms and etiologies associated with many organs (Jones et al., 2025). There are currently 1.3 billion people with high blood pressure worldwide (Kario et al., 2024). During the 2013–2014 period, HTN prevalence in China was highest in the northern province of Liaoning at 37.7% and lowest in the southern province of Hainan at 17.9% (Li et al., 2018). Chinese HTN prevalence has risen, and the highest prevalence in 2021–2022 was found in northern and northeastern regions, with Hebei (38.9%), Inner Mongolia (38.3%), and Heilongjiang (37.8%) showing the highest rates. In the contrast, the provinces with the lowest age-standardized prevalence were located in the southern region (Cao et al., 2025). HTN is influenced by multitudes of factors, including environmental changes, dietary habits, and regional differences, among others. A large multinational analysis demonstrated that exposure to both extreme heat or cold was associated with an increased risk of death from a range of common cardiovascular diseases (Alahmad et al., 2023). A study focusing on low- and middle-income countries had shown that long-term outdoor exposure to PM_2.5_ was linked to an elevated risk of cardiovascular disease in adults aged 35–70 (Hystad et al., 2020).

Dysregulation of the renin-angiotensin-aldosterone system is a key driver in the pathophysiology of HTN. And emerging evidence also revealed the significant contribution of immune cells to this process (Rothman et al., 2020; Bergmark et al., 2019). Gut microbiota plays a huge role in digesting food intake, providing nutrients for the host, and producing a variety of bioactive metabolites. These metabolites not only help to maintain health, but also cause diseases under ecological imbalance conditions (Frolova et al., 2022). A compelling hypothesis is proposed that the gut microbiota serves as a key downstream pathway through both the renin-angiotensin-aldosterone system and the immune system influencing the progression of HTN. So accumulating attention is paid to the effect of intestinal microorganisms on HTN (Li et al., 2017). Mounting evidence has suggested that dysbiosis of the intestinal microbiome is a material environmental element that triggers the onset of HTN. The feces of hypertensive preeclamptic women patients, were transferred to germ-free mouse, and then their blood pressure increased, indicating the mechanism of intestinal biota (Chen et al., 2020; Liu et al., 2019). In recent years, studies have tried to gain insight into gut microbiome interactions in hypertensive patients. A study has found that *S. aureofaciens* Tü117 was present in fecal samples from high-salt diet-induced hypertensive mice and hypertensive patients (Zhou et al., 2025). Some probiotics, such as *Lactobacillus plantarum* CCFM639 attenuates the increase in blood pressure potentially through inhibiting the proliferation of *S. aureofaciens* Tü117 in mice (Zhou et al., 2025). On the other hand, evidences indicate that microbial metabolites also are crucial intermediate factors connected with the intestinal microbiome and the host. Intestinal microbiome plays the role of endocrine organs by producing bioactive metabolites that can directly or indirectly affect the physiological function of the host. Varieties of metabolically active products, including trimethylamine N-Oxide, short-chain fatty acids, primary and secondary bile acids, tryptophan and indole derivatives, and branched-chain amino acids, these may contribute to the cardiovascular disease progression (Tate et al., 2023; Tilves et al., 2022; Lim and van Dam, 2025). Various environmental and host factors contribute to HTN by inducing gut dysbiosis and subsequent microbiota-gut-brain axis dysfunction, which disrupts central blood pressure control mechanisms via immune, metabolic, and neurological pathways (Honarpisheh et al., 2022).

Combined studies of the gut microbiome and metabolome suggested promising prospects for the development of disease control and treatment. Unraveling the interactions between the gut microbiome and metabolome could provide new insights to discover novel targets for the treatment of cardiovascular disease (Sen, 2025). Some studies of the interaction between the human gut microbiome and metabolism in hypertensive patients have already been conducted. Research showed that trimethylamine N-Oxide induces subclinical inflammatory processes involved in cardiovascular disease (Lu et al., 2024). However, some studies mainly focused on common metabolites like trimethylamine N-Oxide, with few researches focusing on the full spectrum of fecal metabolites. And the association between the intestinal microbiome and metabolites has not been comprehensively evaluated in hypertensive patients in different regions.

Daqing (Heilongjiang Province) and Haikou (Hainan Province) are located in the northernmost middle-temperate zone and the southernmost tropical zone of China, respectively. Given the potential differences in gut microbiota and metabolite profiles of hypertensive patients between tropical and temperate zone, this study aimed to characterize the gut microbiome and fecal metabolome in hypertensive patients from these two regions, and to investigate potential microbiota-metabolite features associated with geographic variation.

## 2 Materials and methods

### 2.1 Study population and samples

Conducted from May to December 2024, our study recruited 70 hypertensive patients, including 35 from the Fifth Affiliated Hospital of Harbin Medical University in Daqing, Heilongjiang Province, and 35 from the Department of Cardiology of the Geriatric Hospital of Hainan in Haikou, Hainan Province. The patients were categorized into two groups according to geographic location: the Daqing HTN group (DQ group, *n* = 35) and the Haikou HTN group (HK group, *n* = 35). According to the Chinese Hypertension Prevention and Treatment Guidelines (Joint Committee for Guideline Revision, 2019), the diagnostic standards for HTN are three separate measurements of systolic blood pressure greater than 140 mmHg and/or diastolic blood pressure greater than 90 mmHg while at rest in the non-same day. Each blood pressure measurement ought to be repeated at 1 min interval. In line with these criteria, we enrolled individuals who had been previously diagnosed with HTN and self-reported a history of the condition. Prior to participation in the study, all patients provided written informed consent, as documented in Supplementary File 1. The study protocol received approval from the Ethics Committee of Hainan Medical University (Approval No. HYLL-2020-030). We applied several exclusion criteria across both study groups: recent use of drugs affecting gut microbiota (antibiotics, probiotics, antivirals within 3 months); any underlying chronic disorders (inflammatory, autoimmune, metabolic, or immunodeficient conditions); significant gastrointestinal comorbidity (surgery or active infection within 6 months); pregnancy or lactation; and inability to consent. With the support of volunteers, we collected a morning stool sample from each participant and simultaneously measured their height and body weight to calculate body mass index (BMI). Each sample was split into two tubes or more and then was quickly stored at −80 °C until subsequent processing.

### 2.2 Design of experiment

To investigate the composition of intestinal flora and the metabolite profile among hypertensive individuals across different geographical environments, we conducted a cross-sectional study involving the collection and analysis of fecal samples from hypertensive populations in Daqing and Haikou. Data collected from the two groups included age, sex, height, weight, smoking and drinking status, hypertension history, and admission/discharge diagnoses. Shotgun metagenomic sequencing was conducted on 70 fecal samples to assess their microbiota taxonomic composition. Concurrently, LC–MS metabolomics analysis was performed on these samples to identify a wide array of both established and novel metabolites. The omics analyses were carried out by LC-Bio Technology Co., Ltd. (Hangzhou, China). Following quality control procedures, we excluded 7 fecal samples from the DQ group and 3 fecal samples from the HK group, resulting in the retention of 28 and 32 samples, respectively. Subsequently, we conducted an association analysis between the metagenomic and metabolomic data to identify disease-specific alterations in microbial and metabolic characteristics across different geographical environments, thereby exploring the relationship between diverse microbiota and metabolites.

### 2.3 Gene extraction and metagenomic analysis

Total DNA was extracted from the samples using a genomic DNA extraction kit. DNA libraries were then constructed with the TruSeq Nano DNA Library Preparation Kit (Catalog#FC-121-4001, Illumina, United States) following the manufacturer’s instructions. With a modified step based on the QIAGEN protocol, the total DNA was eluted in 50 μL of elution buffer and stored at −80 °C until PCR analysis was conducted by LC-Bio Technologies Co., Ltd. (Hangzhou, China). Subsequently, metagenomic libraries were sequenced using the Illumina NovaSeq 6,000 platform with PE150 configuration at LC-Bio Technology Co., Ltd. (Hangzhou, China).

Fastp software (version 0.23.4) was employed to remove reads containing adapter contamination, low-quality bases, or undetermined bases. Sequence quality was further verified using Fastp. The remaining high-quality reads from each sample were *de novo* assembled using MEGAHIT (version 1.2.9) and used for microbial taxonomic and functional annotation. Coding sequences of the assembled contigs were predicted using MetaGeneMark (version 3.26), and CDS from all samples were clustered with MMseq2 to generate a non-redundant gene unigenes. Taxonomic profiling of the microbiota was performed using DIAMOND (version 0.9.14) against the NR database. Functional annotation of unigenes, including Gene Ontology and KEGG terms, was also obtained.

Based on the taxonomic and functional annotations of the unigenes, along with their abundance profiles, comparative analyses were performed at the taxonomic and functional levels.

### 2.4 Metabolites extraction and Q-Exactive LC–MS/MS analysis

Collected samples were thawed on ice, and metabolites were extracted with 80% methanol. Briefly, 50 mg of each sample was homogenized with 0.5 mL of pre-cooled 80% methanol, incubated at −20 °C for 30 min, and centrifuged at 20,000 g for 15 min. The supernatant was vacuum-dried and reconstituted in 100 μL of 80% methanol before LC–MS analysis. A pooled quality control sample was prepared by mixing 10 μL of extract from each individual sample. Metabolite profiling was performed on an UltiMate 3,000 UPLC system coupled to a Q-Exactive high-resolution tandem mass spectrometer. Chromatographic separation was carried out on an ACQUITY UPLC T3 column (100 mm × 2.1 mm, 1.8 μm) at 40 °C with a gradient elution program using 5 mM ammonium acetate and 5 mM acetic acid in water (solvent A) and acetonitrile (solvent B) at a flow rate of 0.3 mL/min. Mass spectrometry data were acquired in both positive and negative ionization modes with full-scan MS (m/z 70–1,050) and data-dependent MS/MS using a top-3 method. QC samples were analyzed every 10 injections to monitor analytical stability.

Raw LC–MS data were converted to mzXML format and preprocessed in R using XCMS, CAMERA, and metaX toolboxes, including peak detection, retention time alignment, peak grouping, and annotation of isotopes and adducts, yielding a three-dimensional matrix of retention time-m/z pairs, sample names, and ion intensities. Metabolite annotation was performed by matching accurate molecular masses against the KEGG and Human Metabolome databases with a mass error < 10 ppm. Annotated metabolites were further validated by isotopic distribution and an in-house fragment spectrum library.

### 2.5 Statistical analysis

In this study, SPSS version 20.0 was employed as the primary statistical tool for data analysis, while downstream omics analyses were conducted in R (version 4.1.3). The Shapiro–Wilk test was utilized to assess normality. Continuous variables were compared between groups using the independent-samples *t*-test. Categorical and ordinal variables were evaluated using the Chi-square test, with results expressed as frequencies and percentages. Alpha diversity was quantified using the Chao1, Goods coverage, Shannon, and Simpson indices, while beta diversity was assessed through Principal Coordinates Analysis (PCoA) and Analysis of Similarities (ANOSIM).

Prior to differential analysis, standard preprocessing procedures were applied according to the bioinformatics quality-control pipeline to reduce technical noise. Differential genera between the DQ and HK groups were initially identified using ALDEx2 based on genus-level abundance profiles. The candidate genera identified were subsequently subjected to multivariate linear regression analysis, with adjustment for potential confounding variables including age, BMI, smoking status, and drinking status, to identify genera independently associated with geographic grouping. Following this, a logistic regression model was developed based on genera identified from differential and regression analyses, and its discriminatory efficacy was assessed using receiver operating characteristic curve analysis. For metabolomic analysis, differential metabolites were identified based on a combination of multivariate analysis, Student’s *t*-test, and fold change following data normalization. Multiple testing correction was applied using the Benjamini-Hochberg procedure where appropriate.

All downstream analyses were performed in R (version 4.1.3), including heatmap visualization using the ComplexHeatmap package, volcano plot generation using ggplot2, functional enrichment analysis based on the Reporter Score algorithm, KEGG pathway enrichment analysis of differential metabolites, and co-occurrence network construction using the igraph package. Statistical significance was set at *p* < 0.05.

## 3 Results

### 3.1 Characteristics of the study population

After quality control, 10 samples were excluded, resulting in 60 participants included in the final analysis. The demographic data of the DQ and HK groups was described in Supplementary Table 1. As shown in Table 1, a total of 60 participants were enrolled in our study, comprising 28 in the DQ group and 32 in the HK group. No significant difference was observed in gender distribution between two groups (*p* = 0.569). However, participants in the HK group were significantly older than those in the DQ group (60.44 ± 3.65 years vs. 57.71 ± 4.01 years; *p* = 0.008). Concurrently, the HK group had a significantly lower BMI than the DQ group (22.64 ± 6.73 kg/m^2^ vs. 26.85 ± 3.03 kg/m^2^, *p* = 0.003).

**Table 1:** Demographic characteristics of the DQ and HK groups.

Characteristic	DQ group (*n* = 28)	HK group (*n* = 32)	*P*
Sex, *n* (%)			0.569
Male	18 (64.29)	18 (56.25)	
Female	10 (35.71)	14 (43.75)	
Age (year)	57.71 ± 4.01	60.44 ± 3.65	0.008
BMI (kg/m^2^)	26.85 ± 3.03	22.64 ± 6.73	0.003
Smoking status, *n* (%)			0.028
Yes	9 (32.14)	3 (9.38)	
No	19 (67.86)	29 (90.63)	
Drinking status, *n* (%)			<0.001
Yes	9 (32.14)	0 (0.00)	
No	19 (67.86%)	32 (100.00)	
Frequency of antihypertensive drug use, *n* (%)			0.524
Daily	22 (78.57)	24 (75.00)	
Occasionally	4 (14.29)	3 (9.38)	
NeverNever	2 (7.14)	5 (15.63)	

### 3.2 Species diversity of the gut microbiome

**Figure 1:**
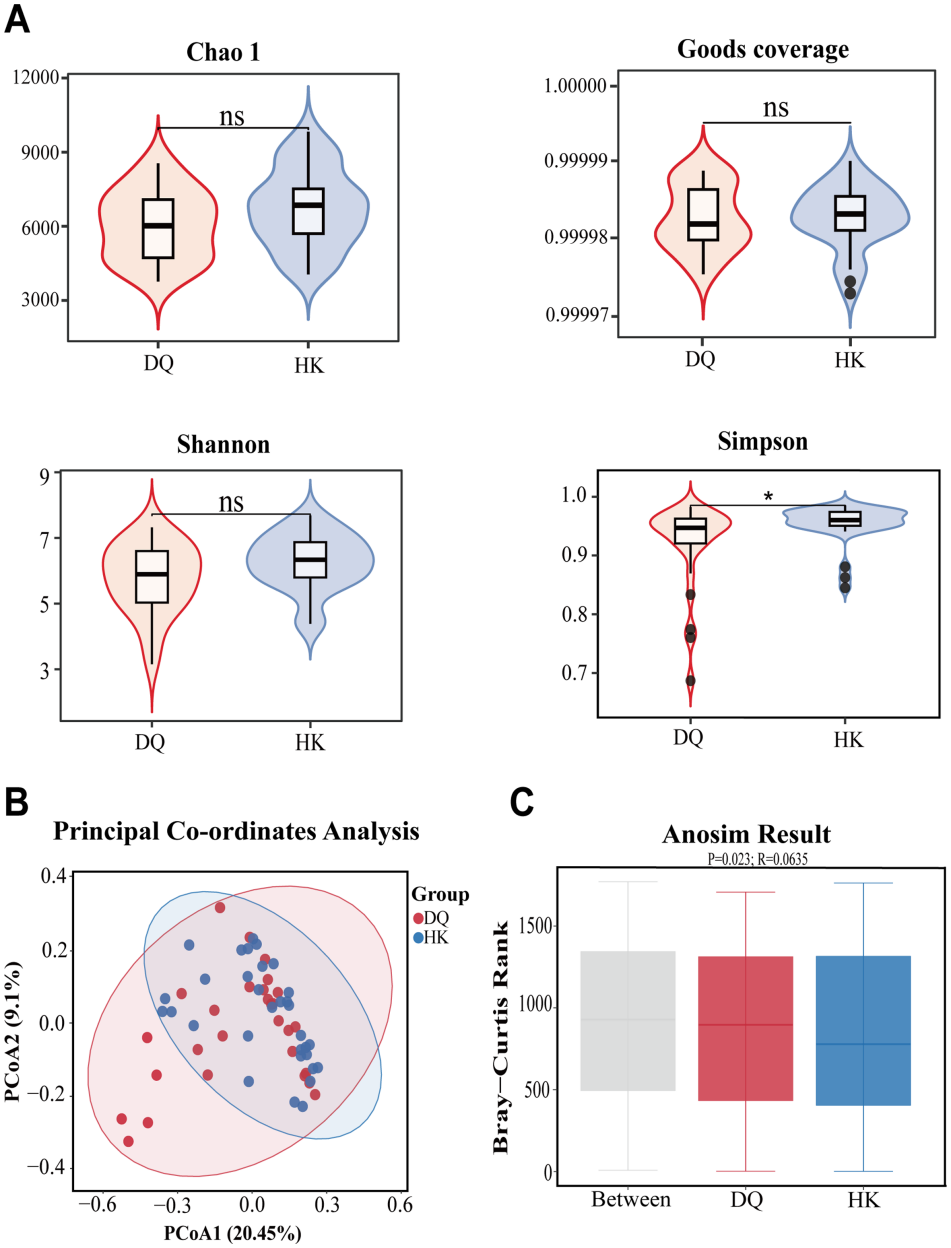
Comparison analysis of species diversity. Alpha diversity was assessed using the Chao1, Goods coverage, Shannon, and Simpson indices. Beta diversity was evaluated using PCoA and ANOSIM. **(A)** No significant differences were observed for the Chao1, Goods coverage, and Shannon index, whereas the Simpson index showed a significant difference. *p < 0.05, **p < 0.01, ***p < 0.001. **(B)** PCoA showed separation between the two groups. **(C)** ANOSIM showed differences in microbial community structure between the DQ group and HK group (*p* = 0.023).

The species accumulation curves of the two groups tended to be stable, indicating that the amount of sequencing data was gradually reasonable, and more data will only produce a small number of new species (Supplementary Figure 1 and Supplementary Table 2). The Chao1, Goods coverage and Shannon indexes of the intestinal flora in the hypertensive group in HK were higher than those in DQ, although there was no statistical significance between the two groups. The Simpson index was significantly different between the two groups (Figure 1A). Although most alpha diversity indices did not reach statistical significance, a trend toward higher diversity was observed in the HK group. PCoA based on Bray–Curtis distance showed a partial separation between the two groups (Figure 1B). ANOSIM was used to inspect the differences between groups and indicated a statistically significant difference (*p* = 0.023); however, the small *R* value (*R* = 0.0635) suggests that the separation between groups was limited (Figure 1C). Taken together, these findings suggest a tendency toward higher microbial diversity in the HK group; however, the overall differences between groups were modest.

### 3.3 Differences in microbial composition associated with the two groups

**Figure 2:**
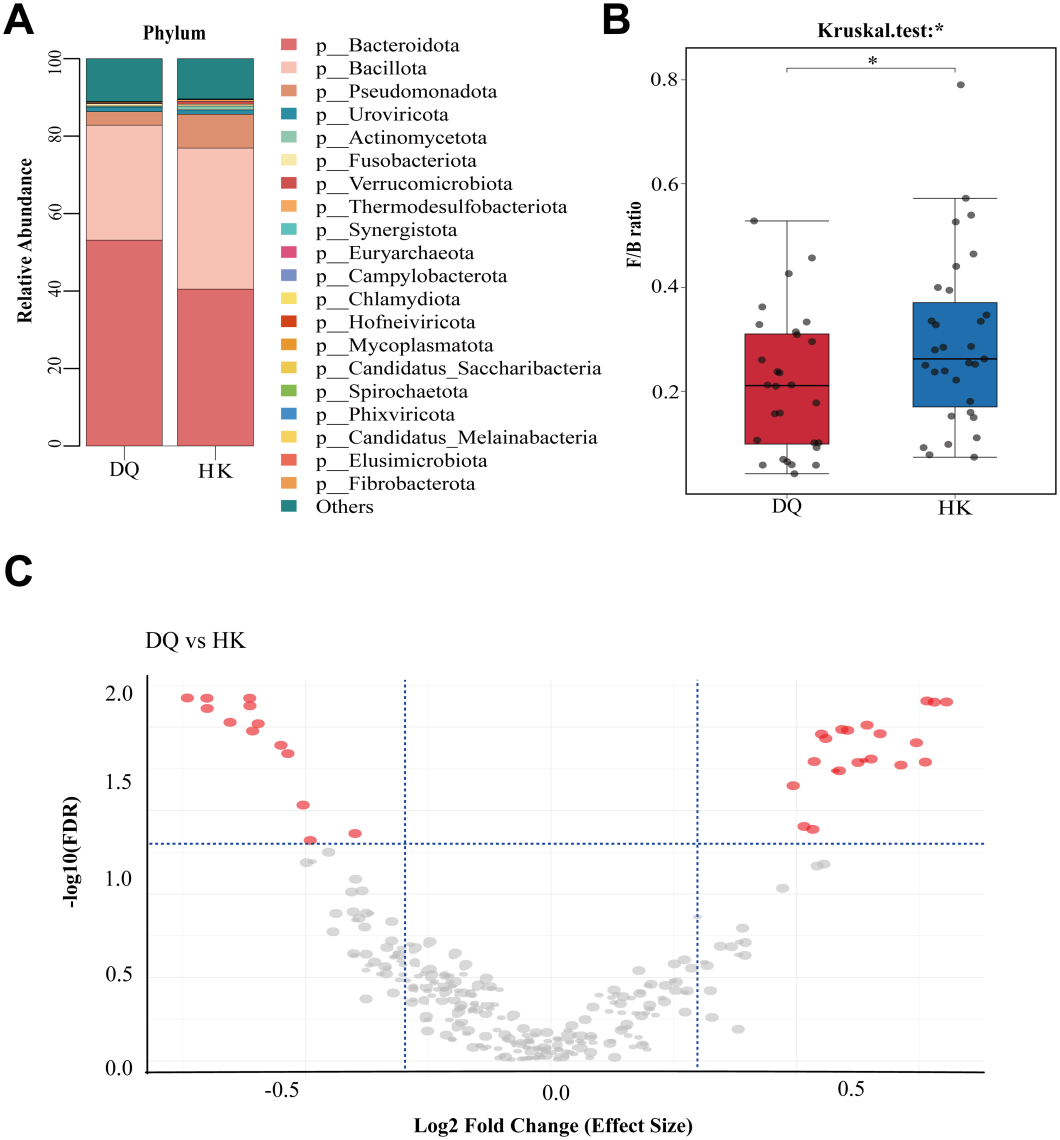
Differential microbial composition between the DQ and HK groups. **(A)** Bar plot showing relative abundance at the phylum level. **(B)** Comparison of F/B ratio between the DQ group and HK group. *p < 0.05, **p < 0.01, ***p < 0.001. **(C)** Volcano plot showing differentially abundant genera identified by ALDEx2 analysis (FDR-adjusted *p* < 0.05).

The differences in the microbial composition between the two groups were first evaluated at higher taxonomic levels. Among the dominant phylum, Bacteroidota was relatively enriched in the DQ group, whereas Bacillota and Pseudomonadota were more abundant in the HK group (Figure 2A). In addition, the Firmicutes/Bacteroidetes (F/B) ratio was significantly lower in the DQ group (*p* = 0.04; Figure 2B).

To obtain more robust and statistically reliable results beyond descriptive comparisons, we performed ALDEx2 analysis at the genus level. A total of 34 genera were identified as differentially abundant between the DQ and HK groups (FDR-adjusted *p* < 0.05; Supplementary Table 3). The distribution of these genera is visualized using a volcano plot (Figure 2C), illustrating both the magnitude of difference (log2 fold change) and statistical significance. Further adjustment for potential confounding factors and downstream analyses are presented in Section 3.4.

### 3.4 The potential value of gut microbiota in distinguishing between the two groups

**Figure 3:**
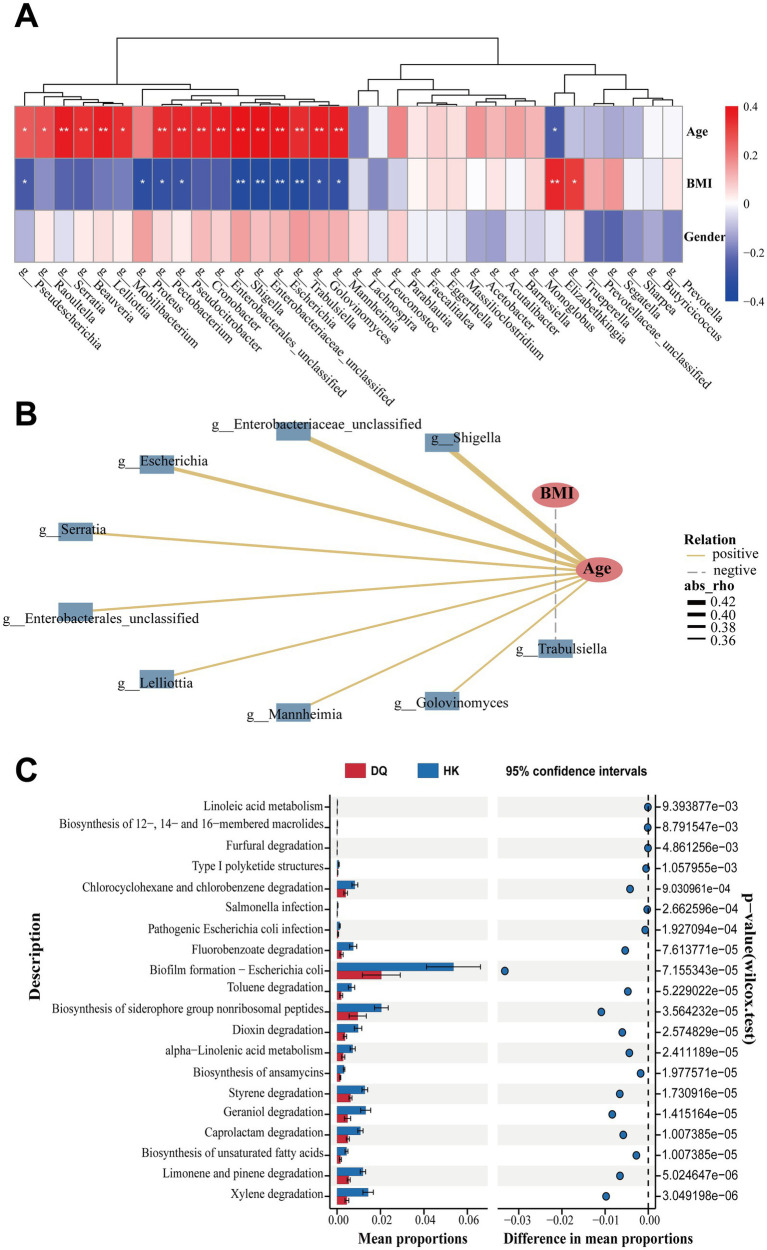
Correlations between gut microbiota and host parameters, along with functional prediction analysis. **(A)** Heatmap revealed correlations between differentially abundant genera and host parameters (Spearman’s correlation; |*r*| ≥ 0.35, *p* < 0.05). **(B)** Age was mainly positively correlated with 8 HK-enriched genera. *p < 0.05, **p < 0.01, ***p < 0.001. **(C)** Top 20 KEGG pathways showing functional differences based on microbiota prediction.

To further evaluate whether the observed microbial differences remain after adjusting for potential confounding factors, we performed additional analyses based on the genera identified through ALDEx2. A total of 34 genera showing differential abundance between the two groups were included in subsequent analyses. Based on Spearman’s correlation analysis, a heatmap was constructed to explore the relationship between these genera and host characteristics (Figure 3A). Several genera enriched in the HK group, including *Shigella*, Enterobacteriaceae_unclassified, *Escherichia*, *Serratia*, Enterobacterales_unclassified, *Lelliottia*, *Mannheimia*, and *Golovinomyces*, showed positive correlations with age (|*r*| ≥ 0.35, *p* < 0.05). Considering that participants in the HK group were significantly older than those in the DQ group, these associations may partly reflect differences in age distribution between the two populations. In addition, BMI was negatively correlated with *Trabulsiella*, while gender showed limited associations with most genera (Figure 3B).

To further account for potential confounding factors, multivariable linear regression analysis was performed using the 34 candidate genera, with adjustment for age, BMI, smoking status, and drinking status. After adjustment, 6 genera (*Elizabethkingia*, *Pseudescherichia*, *Leuconostoc*, *Segatella*, *Serratia*, and Prevotellaceae_unclassified) remained significantly associated with group classification (Supplementary Table 4 and Supplementary Figure 2). These results suggest that part of the observed microbial differences may be independent of baseline demographic variations between the two groups. Based on these genera, a logistic regression model was constructed to evaluate their ability to distinguish between the DQ and HK groups. The model showed acceptable discriminatory performance, with an AUC of 0.8069. Among the individual genera, *Pseudescherichia* showed relatively higher discriminatory ability (AUC = 0.7925; Supplementary Figure 3), suggesting that it may represent a genus associated with geographic differences in gut microbiota profiles.

To further explore potential functional differences, functional profiles were inferred based on gene annotation results from metagenomic sequencing. KEGG pathway analysis identified multiple pathways showing differential enrichment between the two groups (Supplementary Table 5). The top 20 pathways are presented in Figure 3C. Compared with the HK group, pathways such as “Biofilm formation-*Escherichia coli*” and “Biosynthesis of siderophore group non-ribosomal peptides” were relatively reduced in the DQ group. These pathways are predicted to be involved in microbial metabolic and adaptive processes, suggesting potential functional differences in the gut microbiota between the two regions.

### 3.5 Differences in fecal metabolome between the two groups

**Figure 4:**
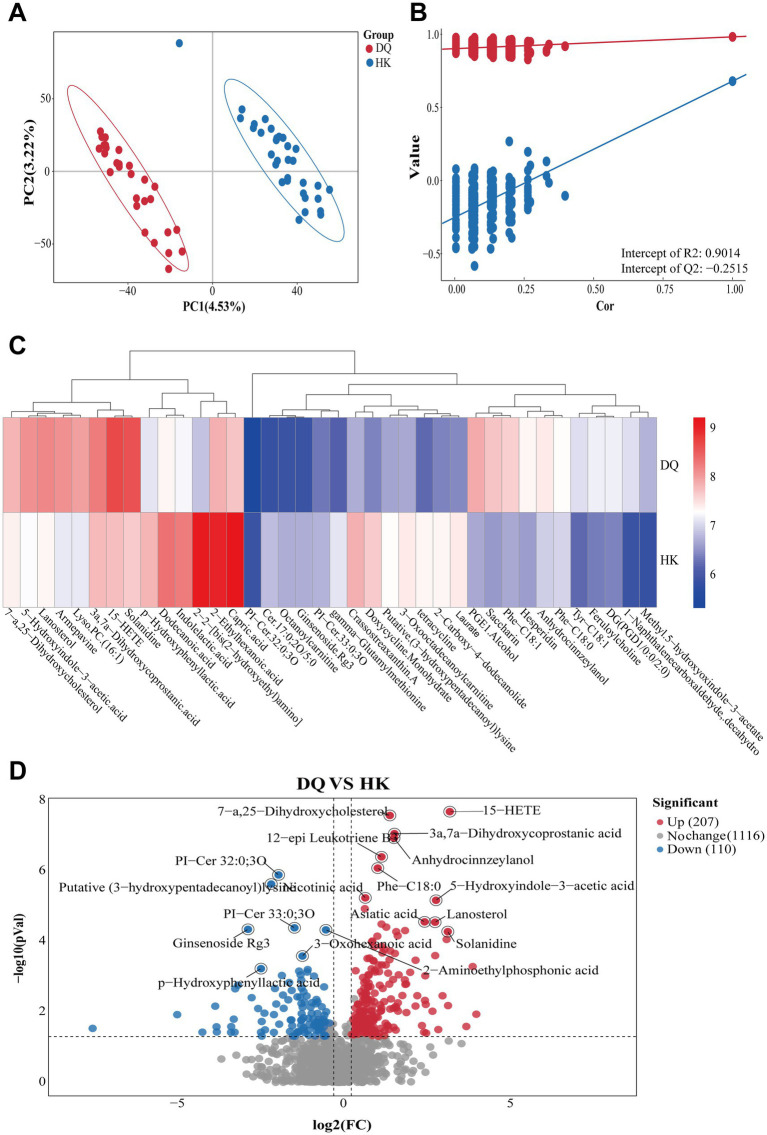
Differences in fecal metabolites between the DQ and HK groups. **(A)** PLS-DA score plot showing separation between groups. **(B)** Model validation based on 200 permutation tests. **(C)** Heatmap showing relative abundance of 38 differential metabolites. **(D)** Differential metabolites identified based on VIP scores, fold change, and statistical significance.

PLS-DA analysis showed a separation between the DQ and HK groups (Figure 4A). Model validation using permutation testing (200 permutations of 7-fold cross-validation) suggested that the model was not overfitted (*Q*^2^ intercept = −0.2515; Figure 4B). A total of 38 differential metabolites were identified between the two groups based on the criteria of VIP ≥ 1, fold change ≥ 1.2 or ≤ 0.83, and *p* < 0.05 (Supplementary Tables 6, 7). The distribution patterns of these metabolites were presented in the heatmap (Figure 4C). Among these metabolites, the DQ group showed relatively higher levels of 15-hydroxyeicosatetraenoic acid (15-HETE), anhydrocinnzeylanol, 7-*α*,25-dihydroxycholesterol, 12-epi leukotriene B3, 3α,7α-dihydroxycoprostanic acid, Phe-C18:0, nicotinic acid, 5-hydroxyindole-3-acetic acid, lanosterol, asiatic acid, and solanidine. In contrast, metabolites enriched in the HK group included PI-Cer 32:0;3O, the putative metabolite (3-hydroxypentadecanoyl) lysine, PI-Cer 33:0;3O, 2-aminoethylphosphonic acid, ginsenoside Rg3, 3-oxohexanoic acid, and p-hydroxyphenyllactic acid. Among these, 15-HETE and 7α,25-dihydroxycholesterol in the DQ group, as well as PI-Cer 32:0;3O and (3-hydroxypentadecanoyl) lysine in the HK group, showed relatively larger differences, and these remained statistically significant after Benjamini–Hochberg adjustment (Figure 4D).

**Figure 5:**
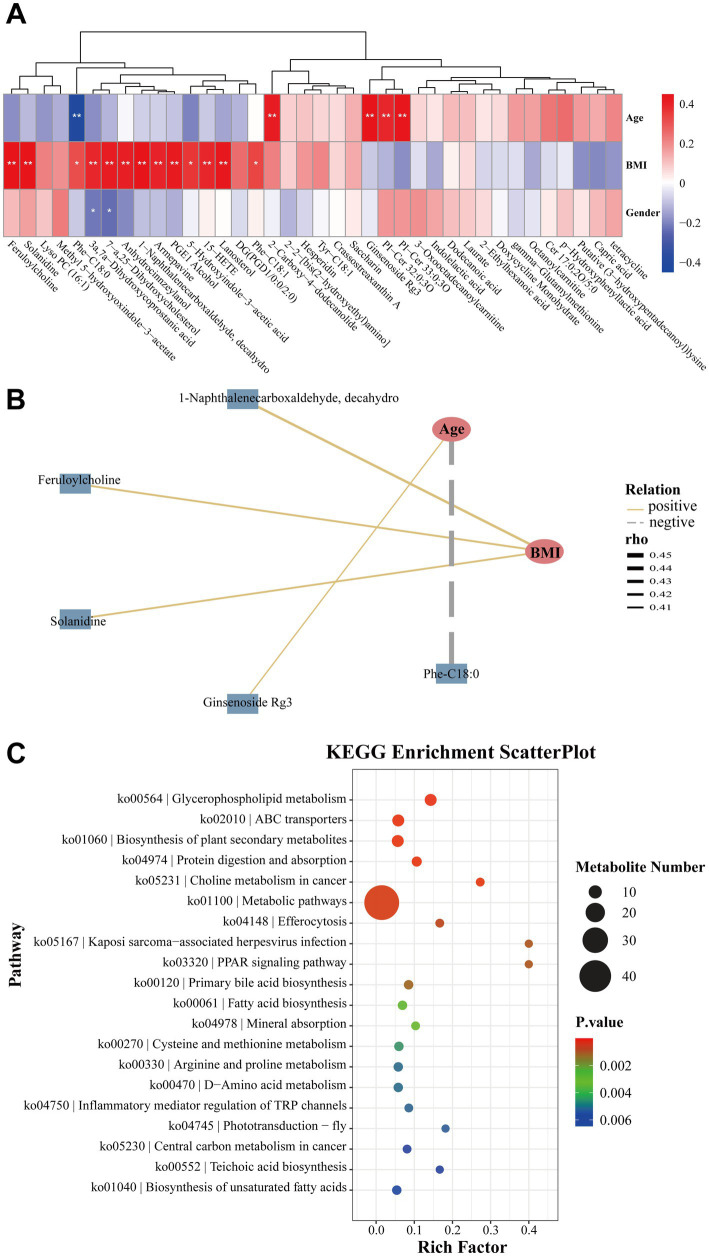
Correlations between fecal metabolites and host parameters, along with pathway analysis. **(A)** Heatmap illustrated the correlations between differential metabolites and host parameters. **(B)** Correlations were subsequently visualized in a co-occurrence network (Spearman’s correlation; |*r*| ≥ 0.4, *p* < 0.05). Age was positively associated with ginsenoside Rg3 and negatively associated with Phe-C18:0, while BMI was positively correlated with 1-naphthalenecarboxaldehyde, decahydro, feruloylcholine, and solanidine. *p < 0.05, **p < 0.01, ***p < 0.001. **(C)** Top 20 KEGG pathways identified from metabolomic enrichment analysis.

To further explore potential associations with host characteristics, Spearman’s correlation analysis was performed. Age showed a positive association with ginsenoside Rg3 and a negative association with Phe-C18:0. In addition, BMI was positively correlated with compounds such as 1-naphthalenecarboxaldehyde, decahydro, feruloylcholine, and solanidine (|*r*| ≥ 0.4, *p* < 0.05; Figures 5A,B). These findings suggest that part of the metabolite variation may be associated with demographic factors such as age and BMI.

KEGG pathway enrichment analysis was then conducted based on the identified differential metabolites. As a result, the top 20 enriched pathways are shown in Figure 5C, and these pathways were identified as significantly altered (false discovery rate < 0.05; Supplementary Table 8). Among these, pathways such as glycerophospholipid metabolism, ABC transporters, biosynthesis of plant secondary metabolites, protein digestion and absorption, choline metabolism, and general metabolic pathways showed differential enrichment between the two groups. Compared with the HK group, several metabolites involved in these pathways were relatively reduced in the DQ group. These pathway-level differences may reflect variations in metabolic activities of the gut microbiota between the two regions. When considered together with the microbial findings described above, the results suggest potential links between gut microbiota composition and metabolite profiles across the two geographic groups.

### 3.6 Correlations between gut microbiota and metabolites

**Figure 6:**
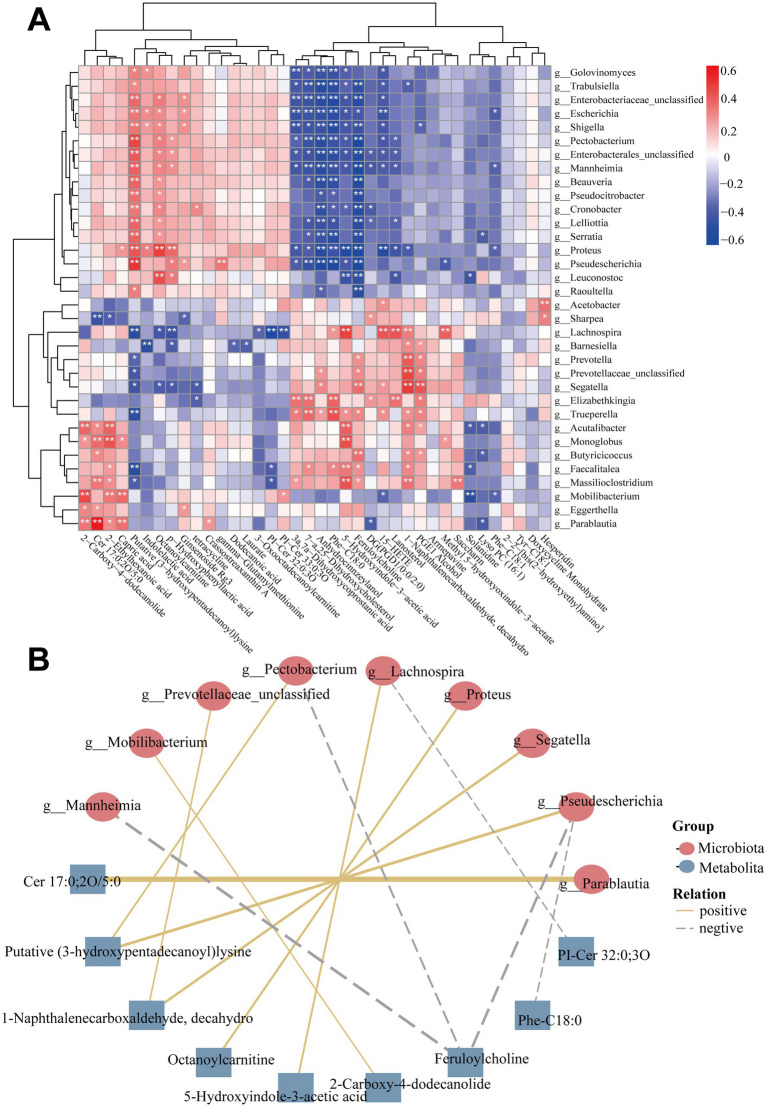
Microbiota–metabolite interactions between the DQ and HK groups. **(A)** Heatmap showing correlations between 34 differentially abundant genera and 38 metabolites (Spearman’s correlation). *p < 0.05, **p < 0.01, ***p < 0.001 **(B)** Co-occurrence network showing microbiota-metabolite correlations (Spearman’s correlation; |*r*| ≥ 0.45, *p* < 0.05). Microbes were represented by circles and metabolites by squares. Yellow lines represent positive correlations, gray lines represent negative correlations, and thicker lines indicate stronger correlations.

To further investigate the microbiota-metabolite interactions between the DQ and HK groups, we evaluated the correlations between 34 genera and 38 metabolites (Figure 6A and Supplementary Table 9). Then a co-occurrence network was subsequently constructed to illuminate the main associations (Spearman’s correlation analysis; |*r*| ≥ 0.45, *p* < 0.05; Figure 6B). In this network, *Pseudescherichia* showed a positive correlation with (3-hydroxypentadecanoyl) lysine, and a negative correlation with feruloylcholine and Phe-C18:0. Moreover, *Parablautia* was positively correlated with the HK-enriched Cer 17:0;2O/5:0. Meanwhile, *Segatella* exhibited a positive association with the DQ-enriched 1-naphthalenecarboxaldehyde, decahydro.

## 4 Discussion

Research on the gut microbiome in patients with HTN across different climatic regions remains limited. In this study, we selected two geographically distinct cities in China, Daqing (middle-temperate) and Haikou (tropical), and applied shotgun metagenomic sequencing combined with untargeted metabolomics to explore microbial and metabolic features associated with regional differences, as well as potential microbiota-metabolite interactions. Compared with the HK group, the DQ group exhibited differences in microbial composition, metabolomic profiles, and microbiota-metabolite associations. In addition, several genera showed potential associations with geographic variation, and a predictive model demonstrated moderate discriminatory performance. These findings suggest potential links between geographic factors and gut microbiota-metabolite interactions in hypertensive patients.

Previous studies on hypertensive patients have reported that the alpha diversity of the gut microbiome is either reduced or remains unchanged (Verhaar et al., 2020). Our findings are consistent with those reports. Furthermore, beta diversity analysis showed differences in microbial community composition between the two groups. At the phylum level, Bacteroidota was relatively enriched in the DQ group, whereas Firmicutes showed a decreasing trend, resulting in a lower F/B ratio compared with the HK group (*p* = 0.04). An increased *F*/*B* ratio is consistently observed in hypertensive patients compared to healthy controls, indicating potential alterations in the gut microbiota (Tsiavos et al., 2024). For example, dietary patterns have been reported to influence the relative abundance of major bacterial phyla, and high-fiber diets may promote the growth of Bacteroidetes (Li et al., 2025). In addition, seasonal variation has been reported to affect gut microbiota composition, with a lower abundance of Bacteroidetes observed in summer, whereas Firmicutes appears less influenced by seasonal changes (Koliada et al., 2020). Taken together, these findings suggest that variations in the F/B ratio between the two groups may be related to differences in environmental or dietary factors, although further studies are needed to clarify these relationships.

Previous studies have reported that members of intestinal microbiota, including *Klebsiella* and *Escherichia*, are more abundant in patients with HTN (Wang et al., 2025; Karki et al., 2024). Consistent with these findings, our results also showed a higher relative abundance of *Escherichia* and *Klebsiella* in fecal samples from the HK group. Members of the Enterobacteriaceae family, including *Klebsiella* and *Escherichia*, have been reported to possess pro-inflammatory properties and may contribute to host immune activation (Xiao and Harrison, 2020). Consistent with previous studies linking HTN to immune activation (Litchman, 2025), the higher abundance of these taxa observed in the HK group may contribute to inflammatory processes. In addition, previous studies have reported that the relative abundance of *Enterobacteriaceae* may vary across different environmental conditions, including seasonal or tropical settings (De Filippo et al., 2010). These findings raise the possibility that alterations in *Enterobacteriaceae* abundance may be related to inflammatory processes observed in HTN. However, given the lack of direct assessment of environmental or dietary factors in this study, these associations should be interpreted with caution.

Following adjustment for confounding factors including age, BMI, smoking and drinking status, the relative abundances of several genera were found to be associated with geographic grouping. Specifically, *Elizabethkingia*, *Segatella* and Prevotellaceae_unclassified were relatively enriched in the DQ group, while *Pseudescherichia*, *Leuconostoc* and *Serratia* were more abundant in the HK group. These distribution patterns may be associated with regional differences, although the underlying factors were not directly assessed in this study. *Segatella* is a member of the Prevotellaceae family, which predominates in the gut microbiota of populations consuming diets rich in grains and plants (Xiao et al., 2024). Previous studies have shown that members of this family are capable of degrading dietary polysaccharides and may be involved in microbial metabolic processes related to fiber utilization (Golisch et al., 2024; Xu et al., 2025). Therefore, the enrichment of *Segatella* and Prevotellaceae_unclassified in the DQ group may reflect differences in dietary patterns or microbial metabolic characteristics. *Elizabethkingia* has been reported in a range of environmental and clinical contexts, but its role within the human gut microbiota remains incompletely understood (Gao et al., 2026). The higher abundance observed in the DQ group may reflect regional variation, although its biological significance requires further investigation. *Serratia* is a genus of Gram-negative bacteria within the Enterobacteriaceae family and has been described as an opportunistic pathogen in certain contexts (Boldeanu et al., 2025). However, its presence and potential role in gut microbial communities, particularly in relation to geographic variation, remain to be further clarified.

In the metabolomic analysis, higher levels of 15-HETE and 7α,25-dihydroxycholesterol were observed in the DQ group. 15-HETE is a product of arachidonic acid metabolism through the 15-lipoxygenase pathway and has been reported to be directly related to vasoconstriction and elevated blood pressure (Szczuko et al., 2020). In addition, 7α,25-dihydroxycholesterol is a ligand for the Epstein–Barr virus-induced gene 2 receptor, which is expressed on activated immune cells and is involved in immune cell migration during inflammatory responses (Nguyen et al., 2024). These findings suggest that these metabolites may be contribute on inflammatory and immune-related processes relevant to HTN. Previous studies have suggested that environmental factors, including cold exposure, may influence blood pressure and lipid metabolic pathways (Sánchez-Gloria et al., 2021). Therefore, the higher abundance of these metabolites in the DQ group may reflect regional differences, although further studies are needed to clarify the underlying mechanisms.

In contrast, several metabolites enriched in the HK group, including PI-Cer 32:0;3O, PI-Cer 33:0;3O, (3-hydroxypentadecanoyl) lysine, and ginsenoside Rg3, have been reported to be associated with cardiovascular and anti-inflammatory processes. PI-Cer serves as a precursor of sphingolipid signaling molecules such as sphingosine-1-phosphate, which is involved in endothelial function and immune regulation (Greiwe et al., 2021). Fatty acid derivatives such as hydroxy fatty acids have been reported to exert anti-inflammatory effects through multiple pathways (Riecan et al., 2022). In addition, ginsenoside Rg3 has been shown to modulate inflammatory signaling and oxidative stress in experimental models (Ren et al., 2021). The enrichment of these metabolites in the HK group may therefore reflect differences in metabolic profiles between the two regions. When considered together with the microbiome findings, these results suggest potential links between gut microbiota composition and metabolite variation across geographic groups.

Alterations in membrane function have been reported to contribute to vascular endothelial dysfunction, altered vascular reactivity, and inflammatory responses in HTN (Liu et al., 2025). In the present study, KEGG pathway analysis revealed differential enrichment of several metabolic pathways between the two groups. For example, pathways related to ABC transporters were relatively enriched in the HK group. ABC transporters have been reported to participate in the transport of a wide range of substrates, including metabolites and xenobiotics, and may be involved in maintaining cellular homeostasis (Duttaroy, 2021). In addition, pathways associated with the biosynthesis of plant secondary metabolites were identified. Such metabolites, including compounds such as ginsenoside Rg3, have been reported to possess anti-inflammatory and antioxidant properties (Redon and Cífková, 2007). In this study, differences were also observed in pathways related to protein digestion and absorption. Previous studies have suggested that these pathways may be influenced by amino acid metabolism and related signaling molecules, including neurotransmitters and vasoactive substances (Maghsoudi et al., 2022). Furthermore, the enrichment of general metabolic pathways may reflect differences in metabolic activity between the two groups, which is consistent with the metabolic characteristics reported in HTN (Ananthakrishnan et al., 2017). In summary, these pathway-level differences may reflect variations in metabolic profiles between the DQ and HK groups.

Although the microbial and metabolite alterations were analyzed separately, they may represent interconnected components of a complex host-microbiota system. Correlation network analysis suggested potential associations between specific microbial taxa and metabolites. For example, a positive correlation was observed between the relative abundance of *Parablautia* and the level of ceramide Cer(17:0;2O/5:0). *Parablautia*, a genus reclassified from *Blautia*, has been reported to be associated with host lipid metabolism in previous studies (Theilmann et al., 2017; Lianqun et al., 2021). These findings may indicate potential links between gut microbiota and lipid-related metabolic processes.

Thus, our study identified differences in the gut microbiota and fecal metabolites in hypertensive patients from middle-temperate and tropical regions of China. However, several limitations should be acknowledged. First, the study only compared hypertensive patient groups from two geographic zones without including a healthy control group. While previous studies have confirmed geographic variations in gut microbiota (Lai et al., 2023; Fan et al., 2025), our design enabled the exploration of location-specific associations within the HTN population. Nevertheless, the extent to which geographic factors specifically influence gut microbiota in individuals with hypertension warrants further investigation in future studies incorporating healthy controls. Second, the sample size was relatively limited, and the analysis was based on a single fecal sample per participant. Future studies should involve larger, multicenter cohorts to validate these findings and identify more representative microbial and metabolic features. To further investigate the impact of environmental factors, such as seasonal variation, longitudinal studies with repeated sampling will be necessary to better understand temporal dynamics and potential causal relationships. Third, the study did not collect detailed data on dietary intake, lifestyle habits, or other relevant factors. These variables are known to be associated with gut microbiota composition and metabolite profiles and may act as confounding factors. The absence of such information may partially limit the interpretation of the relationships among geographic variation, gut microbiota, and metabolites. Future studies should incorporate these variables to improve the robustness and interpretability of the findings.

## 5 Conclusion

This study identified differences in gut microbiota composition and fecal metabolite profiles among hypertensive patients from middle-temperate and tropical regions of China. Variations in microbial diversity and composition were observed between the two groups, along with distinct metabolomic patterns and microbiota-metabolite associations. These findings suggest that geographic variation may be associated with differences in the gut microbiota-metabolite axis in hypertensive populations. Overall, the results provide a basis for further investigations into the potential interactions between gut microbiota and metabolites in HTN. Future research should focus on validating these findings and exploring their underlying biological mechanisms through experimental and longitudinal studies.

## Data Availability

The datasets presented in this study can be found in online repositories. The data is available here: https://www.ebi.ac.uk/ena/browser/view/PRJEB109261. The names of the repository/repositories and accession number(s) can be found in the article/Supplementary material.
