# Supplementary material for: A comparative study of the gut microbiome and fecal metabolome in hypertensive patients from middle-temperate and tropical cities of China: Daqing and Haikou
Source: Front Microbiol. 2026 May 22;17:1801806. doi: 10.3389/fmicb.2026.1801806 (PMC13236899; doi:10.3389/fmicb.2026.1801806)
Supplement: Supplementary file 1 [file Supplementary_file_1.docx]

**Informed Consent Form**

Dear volunteer,

We sincerely invite you to participate in the research project entitled **"** Based on Hainan “migratory birds” population to research molecular mechanism of NAIs regulating hypertension through BER pathway**".** This project has been **reviewed and approved by** the Ethics Committee of Hainan Medical University. It conducts in accordance with the principles of the Helsinki declaration principles and adheres to medical ethics standards. Before you decide whether to participate, please read the following information as carefully as possible. It is designed to help you understand this study. If you wish, you can also discuss it with your family or friends, or ask the researcher for further explanation to assist you to make your decision.

**1.Study Introduction**

**1)Study name:** Based on Hainan “migratory birds” population to research molecular mechanism of NAIs regulating hypertension through BER pathway.

**2)Research purpose:** We order to research the impact and mechanism of environmental change on cardiovascular diseases, providing a reliable theoretical basis for studying how environmental improvement affects cardiovascular diseases in population, and offering scientific evidence for China to strengthen comprehensive air pollution control and public health prevention.

**3)Inclusion criteria:** Individuals were diagnosed with hypertension who voluntarily agree to participate in this experimental study.

**4)Study duration:** May 2024 to December 2024

**2.Researchers’ Qualifications**

Ping zhang, Department of Environmental and Occupational Health, School of Public Health, Hainan Medical University, Professor.

Sha Xiao, Department of Environmental and Occupational Health, School of Public Health, Hainan Medical University, Professor.

**3.Potential Benefits of This Study**

To understand your own cardiovascular health status and the impact of your living environment on health.

To guide people in choosing residential locations with better environmental quality and to improve the prognosis of cardiovascular diseases.

**4.Potential Discomforts and Risks for Volunteers**

Collection of stool samples from volunteers.

**5.Confidentiality**

Your basic personal information will be kept confidential. In the meantime, it may be subject to monitor by relevant authorities (the Ethics Committee) for oversight purposes. These authorities will not disclose your basic personal information to the public.

**6.Contact Information**

**Principal Investigators:** Professor Ping Zhang and Professor Sha Xiao

You can ask any questions regarding this study to Ping Zhang or Sha Xiao, or contact us promptly in case of any emergency.

**7.Volunteer Rights**

You have the right to voluntarily participate in or withdraw from the study, the right to be informed, and the right to give consent. You can withdraw at any time without any loss of benefits or penalties.

**1)Volunteer Participant’ Statement:**

I have carefully read this informed consent form. The researcher has provided me with a detailed explanation and answered all my questions. I fully understand the contents above and agree to participate in the study.

Volunteer Signature: ___________ Date: ___________

**2)** **Researcher Statement:**

I have fully explained and described the purpose, procedure, potential risks and benefits of participation in this study to the volunteer, and have answered all of the volunteer's questions.

Researcher Signature: ___________ Date: ___________
