# Supplementary material for: A comparative study of the gut microbiome and fecal metabolome in hypertensive patients from middle-temperate and tropical cities of China: Daqing and Haikou
Source: Front Microbiol. 2026 May 22;17:1801806. doi: 10.3389/fmicb.2026.1801806 (PMC13236899; doi:10.3389/fmicb.2026.1801806)
Supplement: Supplementary file 2 [file Supplementary_file_2.pdf]

Supplementary Material 1

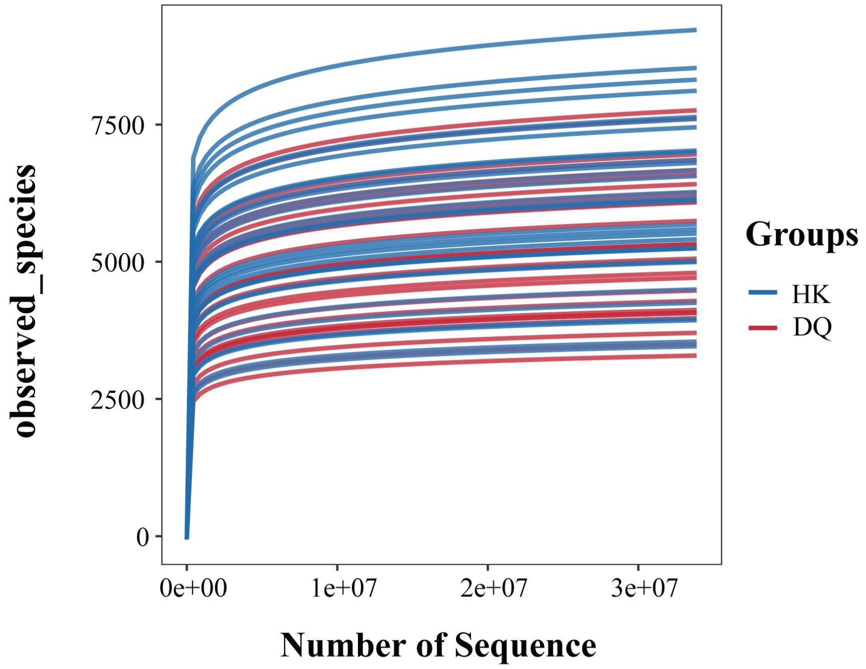

Supplementary Figure 1 The species accumulation curves of the two groups tended to be stable.

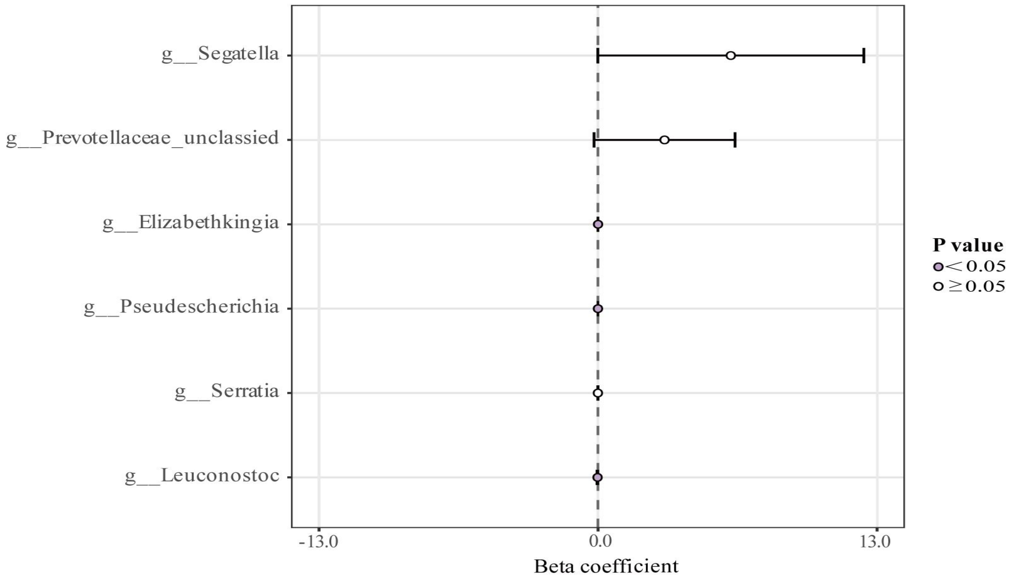

Supplementary Figure 2 6 genera sustained a robust association between the DQ group and the HK group after adjusting for age, BMI, smoking and drinking status.

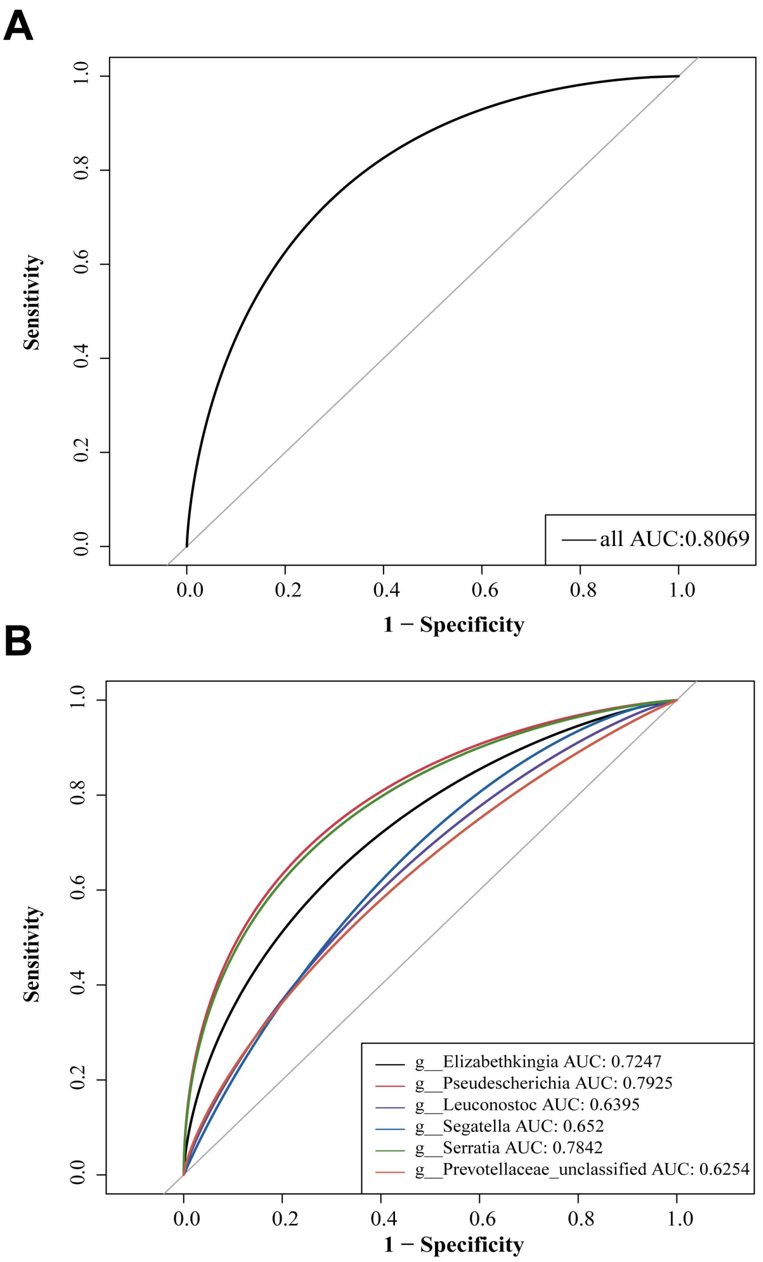

Supplementary Figure 3 Receiver operating characteristic curves evaluating the discriminatory power of 6 geography-associated gut genera between DQ and HK groups. (A) Logistic regression model combining all 6 genera (AUC=0.8069).
(B) Individual genus receiver operating characteristic curves with AUC values labeled in the legend. The gray line represents random classification (AUC=0.5).
